# The anterolateral ligament of the knee joint: a review of the anatomy, biomechanics, and anterolateral ligament surgery

**DOI:** 10.1186/s43019-019-0012-4

**Published:** 2019-11-28

**Authors:** Ji Hyun Ahn, Nilay A. Patel, Charles C. Lin, Thay Q. Lee

**Affiliations:** 1 Department of Orthopaedic Surgery, Dongguk University Ilsan Hospital, 814 Siksadong, Ilsandonggu, Goyangsi, Gyeonggido 411-773 Korea; 2 Department of Orthopaedic Surgery, University of California, Irvine, CA USA; 3 Department of Orthopaedic Surgery, NYU Langone Orthopedic Hospital, New York, New York USA; 4 Orthopaedic Biomechanics Laboratory, Congress Medical Foundation, Pasadena, CA USA

**Keywords:** Anterior cruciate ligament, Knee instability, Anterolateral ligament, ALL reconstruction, Anterolateral tenodesis

## Abstract

Residual knee instability and low rates of return to previous sport are major concerns after anterior cruciate ligament (ACL) reconstruction. To improve outcomes, surgical methods, such as the anatomical single-bundle technique or the double-bundle technique, were developed. However, these reconstruction techniques failed to adequately overcome these problems, and, therefore, new potential answers continue to be of great interest. Based on recent anatomical and biomechanical studies emphasizing the role of the anterolateral ligament (ALL) in rotational stability, novel surgical methods including ALL reconstruction and anterolateral tenodesis have been introduced with the possibility of resolving residual instability after ACL reconstruction. However, there is still little consensus on many aspects of the ALL, including: several anatomical issues, appropriate indications for ALL surgery, and the optimal surgical method and graft choice for reconstruction surgery. Therefore, further studies are necessary to advance our knowledge of the ALL and its contribution to knee stability.

## Background

Anterior cruciate ligament (ACL) reconstruction has improved significantly over the last several decades due to better understanding of anatomy and technical advancements in surgical techniques, resulting in satisfactory results in the majority of cases. Despite these advancements, some patients continue to experience unsatisfactory outcomes with residual knee instability after conventional ACL reconstruction [1]. To address this issue, there has been recent focus on adding additional extra-articular augmentation to ACL reconstruction, specifically with augmentation or reconstruction of the anterolateral ligament (ALL) [2–6]. The ALL is a ligament on the lateral aspect of the knee, anterior to the fibular collateral ligament. Recent anatomical and biomechanical studies have reported on the role of this extra-articular anterolateral structure, demonstrating its synergistic relationship with the ACL with respect to rotational knee stability [2–4]. Despite some arguments against the efficacy of extra-articular ALL reconstruction [7–10], several biomechanical studies have reported that the addition of extra-articular ALL reconstruction showed superior outcomes compared to intra-articular ACL reconstruction alone, especially with regards to objective postoperative knee stability [11–14]. However, there is no consensus on several anatomical issues, including the bony origin and insertion of the ALL, and the change in ALL length with knee flexion [4–6, 15–19]. Due to this, the optimal surgical technique is still debated, with outstanding issues of ideal graft choice [20, 21], location of fixation, and fixation angle [11, 22–24] still unresolved. In the aspect of the surgical indications, the additional ALL surgery is usually recommended for the revision surgery or the ACL-deficient knee with a high-grade pivot-shift test [22, 23]. Recently, its surgical indications have been extended to chronic ACL rupture, concomitant meniscal repair, or pivoting activities [25]. But there is still no consensus for the appropriate surgical indication. The purpose of this review is to highlight the findings of the current literature on the anatomy of the ALL, the function and biomechanics of the ALL, the techniques for ALL surgery, and its clinical outcomes.

## Anatomy

### Prevalence

Among the various names used to refer to this ligamentous structure, such as the “mid-third lateral capsular ligament” and the “capsulo-osseous layer of the iliotibial band (ITB),” the term “anterolateral ligament (ALL)” has been the most widely accepted. Paul Segond, a French surgeon, first reported the presence of the ALL in 1879 [26]. In 2013, Claes et al. further described the presence and characteristics of the ALL [6]. In this study, 41 unpaired human cadaveric knees were examined and the ALL was found as a well-defined ligamentous structure, clearly distinguishable from the anterolateral joint capsule in all but one of the cadaveric knees (97%) [6].

In another cadaveric study by Helito et al. [27], the ALL was found in all dissected anatomical specimens out of 10 specimens (eight knees from men and two from women). Kennedy et al. also reported that they could identify the ALL as a ligamentous structure in all 15 nonpaired, fresh-frozen human cadaveric knees [4]. Daggett et al. reported that the ALL was present in all 52 specimens of embalmed cadaveric knees [18].

However, several anatomical studies did not show 100% prevalence of the ALL. Runer et al. defined the ALL as a ligamentous structure at the anterolateral side of the knee, with a bony origin at the lateral epicondylar region and an oblique course to a bony insertion at the anterolateral proximal tibia [16]. After removing the superficial, deep and capsular-osseous layer of the ITB, the ALL could be clearly identified only in 45.5% (*n* = 20) of the dissected knees according to their definition. Recently, Roessler et al. suggested that the ALL could be identified as an independent ligamentous structure in front of the anterolateral joint capsule in only 60% (*n* = 12) of the dissected knee joints [15].

**Fig. 1:**
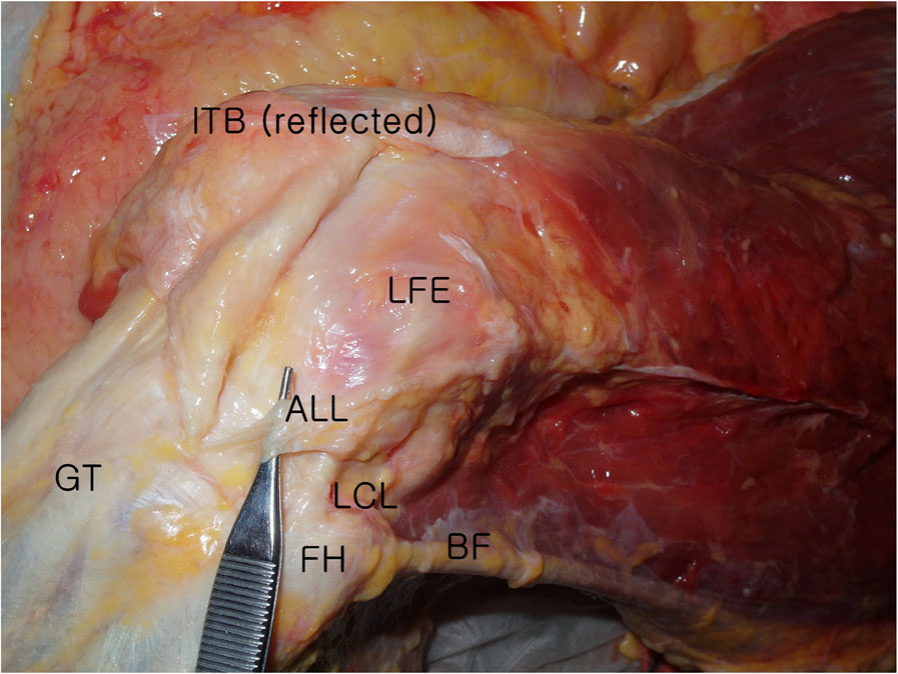
Photograph of dissected specimen. *ALL* anterolateral ligament, *LCL* lateral collateral ligament, *FH* fibular head, *GT* Gerdy’s tubercle, *ITB* iliotibial band, *BF* biceps femoris, *LFE* lateral femoral epicondyle

These previous studies dealing with the presence and prevalence of the ALL [4, 6, 15, 16, 27] have generally used similar dissection protocols to access the ALL. The ITB was sharply detached from the intermuscular septum, and the lateral retinaculum and the fibers were reflected up from their insertion at Gerdy’s tubercle. With the knee flexed at 60° and the tibia maximally internally rotated, the firm fibers running from the lateral epicondyle of the femur to the anterolateral portion of the tibia were unveiled (Fig. 1). Despite the application of similar dissection protocols, the prevalence of the ALL has ranged between 45.5 and 100%. These confusing results could be due to the unclear anatomical definition to distinguish between the ALL and the capsular-osseous layer of the ITB. Helito et al. have even suggested that the ALL consists of two separate layers: the superficial layer located immediately under the ITB and another deeper layer located within the anterolateral capsule [17].

Magnetic resonance imaging (MRI) has been reported as a useful modality to identify the ALL injury in recent studies. It is suggested that MRI on injured knees provides better visualization of the ALL than on intact knees. Soft-tissue inflammation and joint effusion may provide signal intensification, leading to this observation [22, 28]. The assessment of the ALL injury varied between using 1.5-T and 3.0-T MRIs. The recent MRI study suggested that 3.0-T MRI may provide increased visualization [29]. The insertion of the ALL into the proximal tibia just distal to the lateral joint line was well identified in most studies [22, 25, 28–33]. The origin on the distal femur was difficult to visualize because of the close proximity of other lateral structures such as the LCL, popliteus tendon and ITB [22]. The variability in identifying the ALL through the dissections in previous anatomical studies may also explain the various results in the identification of the ALL injury in MRI studies. Monaco et al. reported that MRI is highly sensitive, specific, and accurate for the detection of abnormalities of the ALL and anterolateral capsule and shows a high percentage of agreement with surgical findings [30]. They proved that the percentage agreement between MRI and surgical findings was 88% for ALL and anterolateral capsule injuries through the surgical exploration in acute ACL-injured knees. In a recent systemic review, the ALL appeared on the MRI findings in 51–100% of all assessed 2427 knees in a total of 24 studies [28]. This study suggested that high variability was found in the identification of normal and injured ALL in MRI, and the entire portion of the ligament was often not seen.

### Attachment site of the ALL (Table 1)

Although there have been various anatomical studies of the ALL, there is still controversy regarding its anatomical parameters. The most frequently seen difference among previous studies is the femoral attachment site of the ALL, with various descriptions of anterior and distal, in the center, or posterior and proximal to the lateral epicondyle of distal femur [4–6, 15–19, 27, 34, 35]. In the initial anatomical study by Claes et al., the major femoral attachment of the ALL was located at the prominence of the lateral femoral epicondyle, slightly anterior to the origin of the lateral collateral ligament [6]. Conversely, Dodds et al. reported that the ALL passed antero-distally from its femoral attachment, proximal and posterior to the lateral femoral epicondyle to the margin of the lateral tibial plateau, approximately midway between Gerdy’s tubercle and the head of the fibula [5]. Similar to the controversy in these two earlier studies, a variety of reports have been made in later studies on whether the bone attachment site is anterior and distal, or posterior and proximal to the femoral lateral epicondyle [4, 16, 18, 34]. In a recent study, it was stated that the superficial ALL was located posterior and proximal to the lateral epicondyle, while the deep ALL was located in the center of the lateral epicondyle [17].

**Table 1:** Attachment site and length change of the anterolateral ligament

	Femoral attachment	Tibial attachment	Length change
Claes et al. (2013) [6]	Prominence of LFE or anterior to LFE	21.6 ± 4.0 mm posterior to GT, 23.2 ± 5.7 mm anterior to FH	41.5 ± 6.7 mm in 90° of flexion, 38.5 ± 6.1 mm in extension (*p* < 0.001)
Dodds et al. (2014) [5]	4.3 ± 4.9 mm posterior, 8.0 ± 5.2 mm proximal to LFE	18 ± 3 mm posterior to GT, 17 ± 3 mm anterior to FH	Isometric from 0 to 60° of (1.7 ± 1.1 mm, *p* = 0.980), shortening of 4.1 ± 0.9 mm (*p* = 0.011) from 60 to 90°.
Kennedy et al. (2015) [4]	Posterior and proximal to LFE with the distance of 7.0 mm (5.6–8.4)	24.7 mm posterior to GT, 26.1 mm anterior to FH	A continuous increase in length with increasing knee flexion: 41.6 mm in 90° of flexion, 36.8 mm in extension
Zens et al. (2015) [19]	(none)	(none)	Continuous increase in length with increasing knee flexion: 10.15% per degree (*p* < 0.001)
Runer et al. (2016) [16]	LFE (45.0%) or just posterior and proximal to LFE (55.0%)	18.6 ± 3.8 mm posterior to GT, 15.2 ± 3.9 mm anterior to FH	Lengthening from 0 to 60° (4.7 ± 2.5 mm, *p* < 0.001), of shortening of 1.0 ± 1.6 mm (*p* = 0.015) from 60 to 90°
Dagget et al. (2016) [18]	12 (23%): directly to LFE, 30 (58%): slightly proximal and posterior to LFE, 10 (19%): completely proximal and posterior to LFE	(none)	(none)
Kosy et al. (2016) [34]	1: directly to LFE, 6: proximal and posterior to LFE, 3: distal and anterior to LFE	17.7 ± 2.95 mm posterior to GT, 12.3 ± 3.55 mm anterior to FH	(none)

*LFE* lateral femoral epicondyle, *GT* Gerdy’s tubercle, *FH* fibular head

## Biomechanics

### Length change of the ALL

Due to the uncertain femoral attachment, the length change according to the knee flexion angle has not yet been determined. In previous cadaveric studies, there has been the different description of length-change patterns of the ALL during knee flexion. Dodds et al. observed the ALL to be close to isometric between 0 and 60° of knee flexion and decreased in length from 60 to 90° of flexion [5]. These findings are in direct contrast to previous studies [9, 16, 19, 27] which found the ALL to be nonisometric and to gradually increase in length during 0 to 90° of flexion. Imbert et al. found that the length change of the ALL was dependent on how its femoral attachment site was defined [35]. Length variations referencing three different anatomical femoral insertions of the ALL (at the center of the lateral epicondyle, distal and anterior from this position, and proximal and posterior) demonstrated a decrease in length with the proximal-posterior position but an increase in length for both the epicondyle and the distal-anterior location. Additionally, with the concept of the ALL composed of two distinct structures, Helito et al. [17] reported that the length of the superficial ALL increased with knee extension, and the length of the deep ALL increased with knee flexion. These results are important when considering the optimal knee-flexion angle and location of femoral tunnel placement for graft fixation during ALL reconstruction.

### Function of the ALL

Various previous biomechanical studies have demonstrated a significant effect of the ALL in providing rotational stability to the knee [2, 11, 14, 22, 23, 36–38]. Nitri et al. [14] suggested that ACL reconstruction with ALL deficiency had significant increases in internal rotation compared to both the intact knee and ACL reconstruction with ALL-intact conditions during simulated pivot shift. In another cadaveric study by Rasmussen et al [29, 38]. combined sectioning of the ACL and ALL resulted in a significant increase in axial-plane tibial translation during a simulated pivot shift, when compared with ACL-only sectioning. From this result, they suggested that residual internal rotation and a positive pivot shift after ACL reconstruction may be attributed to ALL injury. Sonnery-Cottet et al. also reported the involvement of the ALL in rotational control of the knee at varying degrees of knee flexion [2]. After ACL sectioning, an incision of the ALL induced a significant increase in internal rotation at 20° and at 90°, and in simulated pivot shift at 30°. Tavlo et al. [37] reported that detaching the ALL had a significant effect not only on internal rotatory stability and but also on anterior-posterior stability in ACL-insufficient knees.

In contrast, several studies have reported a limited role of the ALL in rotational knee stability [7–10]. Noyes et al. suggested that in their cadaveric study, although the ALL reconstruction corrected small abnormal changes at the limit of internal rotation at high flexion angles (within 0.5° and 0.7° of the ACL-reconstructed state at 60° and 90° of flexion, respectively), the procedure had no effect in limiting tibiofemoral compartment translation in the pivot-shift test [8]. With the small changes in rotational stability after ALL sectioning, they suggested that the recommendation to perform an ALL reconstruction to correct pivot-shift abnormalities was questionable. Overall, considerable controversy regarding the function of the ALL remains.

### Effect of ALL surgery

Among the recent biomechanical studies on anatomical ALL reconstruction [7, 8, 10, 14, 37], there have been several studies that have questioned the efficacy of ALL reconstruction [7, 8, 10]. Stentz-Olesen et al. [7] reported that reconstructing the ALL using a gracilis autograft tendon did not decrease the internal rotation laxity in the ACL-reconstructed knee. Based on the results of this study, they did not recommend reconstructing the ALL in ACL-reconstructed knees for the purpose of decreasing internal knee laxity [7]. Schon et al. reported that anatomical ALL reconstruction at all graft fixation angles from 0 to 90° significantly overconstrained internal rotation of the knee joint [10]. However, Nitir et al. and Tavlo et al. suggested that combined anatomical ALL and ACL reconstructions significantly improved the rotatory stability of the knee compared to isolated ACL reconstruction when there was concurrent ALL deficiency [14, 37]. When single-bundle ACL reconstruction with anterolateralplasty was compared to double-bundle reconstruction using cadaveric ACL/ALL knees [13], Bonanzinga et al. reported that internal rotation and the pivot-shift test were better controlled by single-bundle reconstruction with anterolateralplasty compared to the double-bundle ACL reconstruction at both 30 and 90° of flexion. However, a dated technique for intra-articular reconstruction with graft placement over the top of the lateral femoral condyle was used. Therefore, these results may be misinterpreted by surgeons who may underestimate the outcomes of intra-articular double-bundle ACL reconstruction.

## Surgery of the ALL

### Surgical techniques

**Fig. 2:**
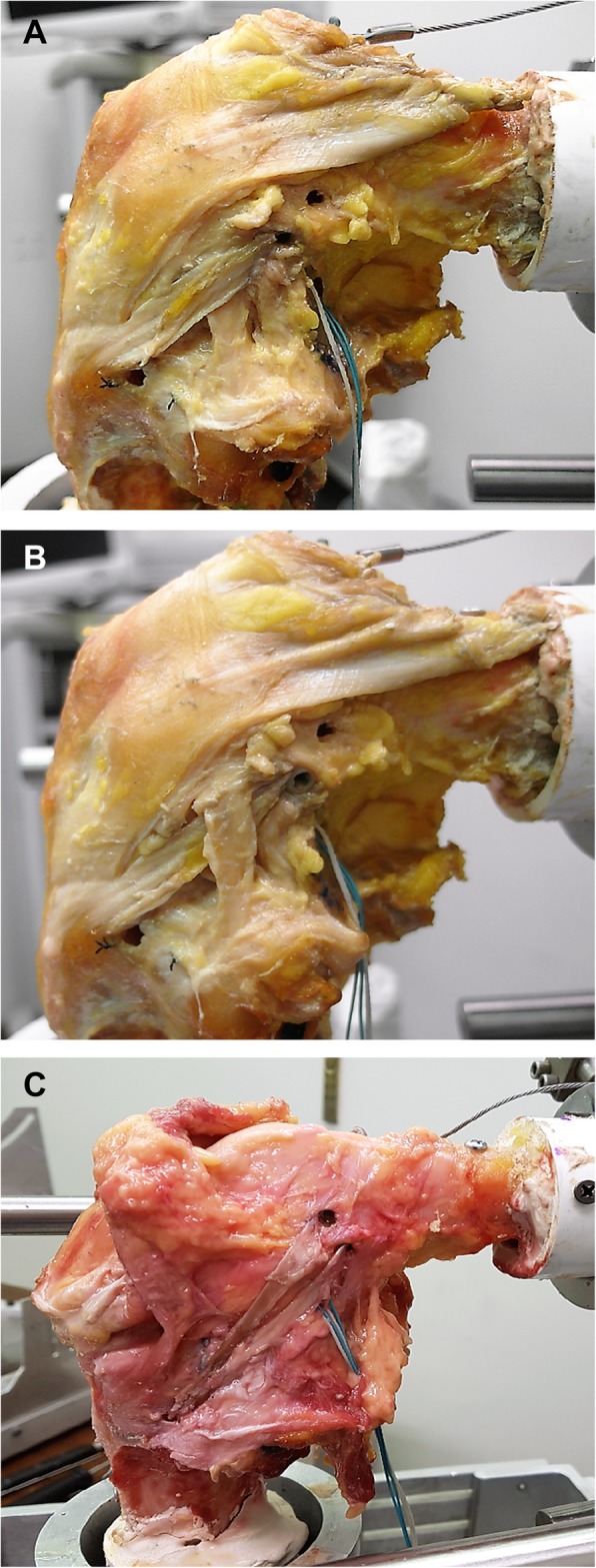
Modified Lemaire tenodesis passed superficial (**a**) and deep (**b**) to the lateral collateral ligament using the iliotibial band in a cadaveric knee, and anatomical anterolateral ligament (ALL) reconstruction using the gracilis tendon (**c**)

For ALL reconstruction, the ideal graft to use and the optimal degree of knee flexion at which to fix the graft have not been clearly established [22, 23]. Additionally, many different surgical techniques have been described, including non-anatomical anterolateral tenodesis (Fig. 2a and b) and anatomical ALL reconstruction (Fig. 2c) [22, 23, 39].

Several studies have suggested that anterolateral augmentation with the ITB could be an effective surgical method to reduce residual internal rotation and a positive pivot shift after ACL reconstruction [3, 11, 39]. To biomechanically compare various extra-articular anterolateral surgeries, Inderhaug et al. performed ACL reconstruction alone and in combination with the following: modified MacIntosh tenodesis, modified Lemaire tenodesis passed both superficial and deep to the lateral collateral ligament, and anatomical ALL reconstruction with 20 N and 40 N of graft tension [11]. In this study, the modified MacIntosh tenodesis was performed using a central strip of the ITB. The graft was routed deep to the LCL and fixed into the bone tunnel positioned 70 mm proximal to the femoral epicondyle. In the modified Lemaire tenodesis, the central strip of the ITB was routed deep to the LCL and fixed into a bone tunnel positioned proximal and slightly posterior to the lateral epicondyle. In the combined ACL plus anterolateral-injured knee, ACL reconstruction alone failed to restore intact knee kinematics when an anterior drawer force and internal torque was applied. The deep Lemaire and MacIntosh procedures restored rotational kinematics to the intact state, while the anatomical ALL reconstruction underconstrained internal rotation and the superficial Lemaire overconstrained internal rotation [11].

There is still no consensus on the optimal graft choice for ALL reconstruction [11, 20, 21]; however, autogenous ITB has been mainly used for extra-articular tenodesis, while autogenous gracilis grafts have been mainly used for anatomical ALL reconstruction. In a biomechanical study regarding graft properties during ALL surgery, Wytrykowski et al. reported that the gracilis (200.7 N) had a significantly higher failure load than ITB (161.1 N) and ALL (141 N) [20]. Therefore, they suggested that the ITB’s mechanical properties most closely resembled the ALL.

### Clinical outcomes

For the past 5 years, there has been a paucity of data regarding clinical outcomes after simultaneous extra-articular ALL reconstruction during ACL surgery [12, 40–45]. Only a few studies [40, 41, 43, 44] have been done to compare combined ACL/ALL reconstruction and isolated ACL reconstruction.

In a prospective comparative study between combined reconstruction of both the ACL and ALL versus isolated anatomical reconstruction of the ACL [43], at a mean final follow-up of 27 months, none of the patients (*n* = 0: 0.0%) who underwent combined ACL and ALL reconstruction had anterior translation of greater than 5 mm at maximum pulling strength compared with their normal knees. Conversely, three (6.0%) patients who underwent isolated ACL reconstruction had anterior translation of more than 5 mm. Surgical indication for the combined reconstruction of the ACL and ALL included the following criteria: grade 2 pivot-shift, high level of the sporting activity, participation in pivoting sports, chronic ACL injury, or Segond fracture. These findings were not significantly superior to isolated ACL reconstruction, therefore Ibrahim et al. recommended that ALL reconstruction should not be performed routinely for patients undergoing ACL reconstruction [43].

In another prospective comparative study by Sonnery-Cottet et al. [41], patients underwent primary ACL reconstruction with a bone-patellar tendon-bone (B-PT-B) graft, quadrupled hamstring tendon (4HT) graft, or hamstring-tendon graft combined with ALL reconstruction. This study included all young patients (aged 16–30 years) who were participating in pivoting sports before injury. At a minimum follow-up of 2 years, the rate of graft failure with HT + ALL grafts was 2.5 times less than with B-PT-B grafts and 3.1 times less than with 4HT grafts. The patients who had the HT + ALL graft had greater odds of returning to preinjury levels of sport when compared with the patients with 4HT graft (odds ratio (OR), 1.938; 95% CI, 1.174–3.224).

In a retrospective comparison of single-bundle ACL reconstruction [44], double-bundle ACL reconstruction, and combined single-bundle ACL and ALL reconstruction, the postoperative knee stability and joint functions of the double-bundle ACL-reconstruction group and the combined single-bundle ACL-reconstruction and ALL-reconstruction group were better than the isolated single-bundle reconstruction group. No significant difference was observed between the double-bundle reconstruction group and the combined single-bundle and ALL reconstruction group. The inclusive criteria were that all patients were nonprofessional athletes and non-heavy manual workers with sports injury or injury caused by light violence in daily life. Most patients were identified as grade II on the Lachman test and grade I on the pivot-shift test, preoperatively.

Recently, Lee et al. reported the comparative study to assess the effect of the ACL reconstruction in combination with ALL reconstruction on revision ACL reconstruction [40]. They suggested that revision ACL reconstruction in combination with ALL reconstruction significantly reduced rotational laxity and showed a higher rate of return to the same level of sports activity than isolated revision ACL reconstruction alone, although there were no significant differences in anterior laxity or functional test results between the two groups.

## Conclusion

Many questions still remain regarding the anatomy and function of the ALL. There is no consensus on several anatomical issues of the ALL including the anatomical bony origin and changes in length with knee flexion. These two anatomical issues are essential to establish the surgical procedure for ALL reconstruction or anterolateral tenodesis. It is still unclear whether the reconstruction is clinically effective, despite positive suggestions from recent biomechanical studies. Additional anatomical and biomechanical studies are required to better define the optimal surgical technique. Furthermore, comparative clinical studies with long-term follow-up are needed to evaluate the clinical efficacy of ALL reconstruction.

## Data Availability

Not applicable.

## Abbreviations

4HT: Quadrupled hamstring tendon
ACL: Anterior cruciate ligament
ALL: Anterolateral ligament
B-PT-B: Bone-patellar tendon-bone
ITB: Iliotibial band
OR: Odds ratio
